# A novel fluorescent off–on probe for the sensitive and selective detection of fluoride ions†

**DOI:** 10.1039/c9ra06342k

**Published:** 2019-10-10

**Authors:** Lihong Li, Min Zhang, Kaijing Chang, Yu Kang, Guodong Ren, Xiaoyu Hou, Wen Liu, Haojiang Wang, Bin Wang, Haipeng Diao

**Affiliations:** Department of Biochemistry and Molecular Biology, Shanxi Medical University Taiyuan 030001 PR China lilh@sxmu.edu.cn diaohp@sxmu.edu.cn; School of Basic Medical Sciences, Shanxi Medical University Taiyuan 030001 PR China

## Abstract

A highly sensitive and selective fluorescent probe for fluoride ions has been developed by incorporating the dimethylphosphinothionyl group as a recognition moiety into the fluorophore of coumarin. The detection mechanism is based on the fluoride ion-triggered cleavage of the dimethylphosphinothionyl group, followed by the release of coumarin, which leads to a large fluorescence enhancement at 455 nm (*λ*_ex_ = 385 nm). Under the optimized conditions, the fluorescence enhancement of the probe is directly proportional to the concentration of fluoride ions in the range of 0–30 μM with a detection limit of 0.29 μM, which is much lower than the maximum content of fluoride ions guided by WHO. Notably, satisfying results have been obtained by utilizing the probe to determine fluoride ions in real-water samples and commercially available toothpaste samples. The proposed probe is rather simple and may be useful in the detection of fluoride ions in more real samples.

## Abstract 2

A sensitive and selective fluorescent off–on probe is developed for fluoride ion detection, and its applicability has been demonstrated by determining fluoride ions in real-water samples and toothpaste samples.

## Graphical abstract

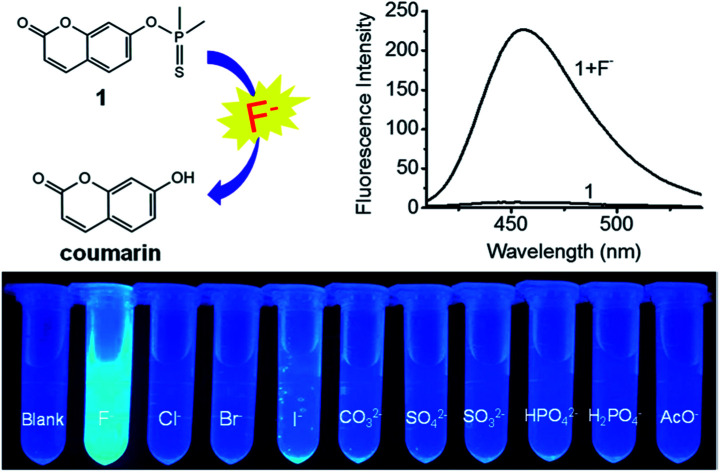

## Introduction

Fluoride ions (F^−^), as the smallest anion with the highest electronegativity, play a major role in health and environmental science. Appropriate intake of F^−^ has proven to be critical for preventing tooth cavities and treating osteoporosis.^1–3^ Thus water fluoridation or addition of fluoride to toothpaste has been extensively employed in a great many countries.^4,5^ However, the excess ingestion of F^−^ may lead to fluorosis, accompanied with a series of diseases, such as tooth mottling, kidney and gastric disorders, metabolic disturbances, neurotoxicity and so on.^6–8^ Therefore the amount of F^−^ in water and other fluoride supplements must be strictly limited. As suggested by the World Health Organization (WHO), the maximum level of F^−^ in drinking water should be 1.5 mg L^−1^.^9^ Unfortunately, fluoride contamination in groundwater, which means the F^−^ concentration is higher than 1.5 mg L^−1^, has been regarded as a severe threat throughout the world.^10^ Therefore, it is of great importance to develop a simple approach to monitor fluoride ions in water as well as other samples.

Among the present analytical methods for F^−^, fluorescent probes have caught great attention due to their high sensitivity and selectivity as well as easy operation.^11–15^ To date, fluorescent probes based on various sensing mechanisms have been reported for the detection of F^−^, including the fluoride–hydrogen bonding interaction,^16,17^ boron–fluoride complexion^18,19^ and fluoride induced chemical reaction.^20–32^ Among them the first two types of probes could only work in non-aqueous media or media with high percentage of organic solvent because the detection signal would be largely hindered in aqueous environment.^23,24^ So the third strategy, that is fluoride induced chemical reaction, has attracted much attention. Owing to the high affinity of F^−^ towards silicon, several probes have been developed relying on the fluoride-triggered cleavage of Si–O bond.^20–22,24–30,32^ Besides, dimethyl-phosphinothionyl groups, which have been utilized as the protecting groups for side chain phenolic OH of tyrosine in peptide synthesis, could also be easily removed by F^−^.^33,34^ Thus dimethylphosphinothionyl group would be a potential recognition moiety for F^−^. However, fluorescent probes employing such a recognition unit are still rare.^23,31^ Herein, we report probe 1 as a sensitive and selective fluorescence off–on probe for F^−^.

**Scheme 1:**
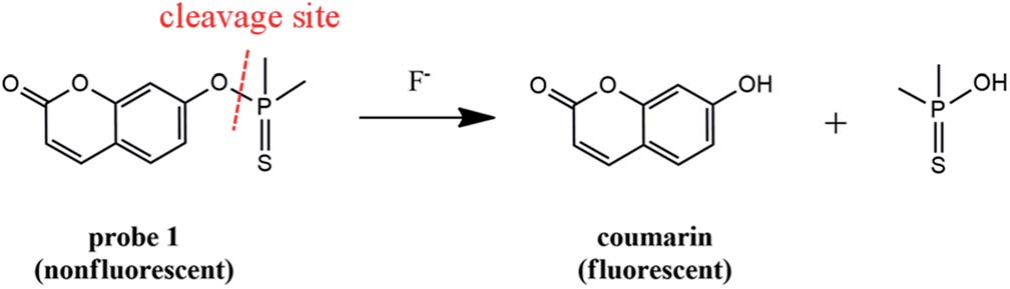
Structure of probe 1 and its reaction with F^−^.

The probe (Scheme 1) is designed by incorporating the specific recognition moiety (dimethylphosphinothionyl group) into coumarin. Probe 1 itself is nearly nonfluorescent due to the hydroxyl blocking of coumarin,^35–37^ which is favourable for achieving high detection sensitivity. Reaction of 1 with F^−^ causes the cleavage of the P–O bond between dimethylphosphinothionyl group and coumarin, accompanied by the release of coumarin and dramatic fluorescence enhancement, which results in the establishment of a sensitive and selective method for assaying F^−^. The probe has been successfully applied to determine F^−^ in real-water samples and commercially available toothpaste samples.

## Experimental

### Reagents and apparatus

7-Hydroxyl-coumarin, dimethylthiophosphinoyl chloride and triethylamine were purchased from Sigma-Aldrich. NaF, NaCl, KBr, NaI, Na_2_CO_3_, Na_2_SO_4_, Na_2_SO_3_, K_2_HPO_4_ and KH_2_PO_4_ were purchased from Beijing Chemicals, Ltd. Sodium acetate and tris(hydroxymethyl)aminomethane (Tris) were obtained from J & K Chemical Ltd. Ultrapure water (over 19 MΩ cm) from a Smart-N system (Heal Force, China) was used throughout.

^1^H- and ^13^C-NMR spectra were performed on a Brucker DMX-400 spectrometer. High resolution electrospray ionization mass spectra (HR-ESI-MS) were operated on an APEX IV FTMS instrument (Bruker Daltonics). UV-Vis Absorption spectra were measured with a UV-6100 spectrophotometer (Mapada, China) in 1 cm quartz cells. Fluorescence spectra were collected on a Varian spectrofluorimeter (Cary Eclipse, America) in 1 × 1 cm quartz cells.

### Synthesis of probe 1

As shown in Scheme S1†, probe 1 was synthesized *via* a one-step method. In brief, triethylamine (88 μL, 0.63 mmol) was added to a solution of 7-hydroxyl-coumarin (40.5 mg, 0.25 mmol) in dry dichloromethane (15 mL), followed by the addition of dimethylthiophosphinoyl chloride (64.3 mg, 0.5 mmol). Then, the mixture was stirred at ambient temperature for 12 h. After removal of the solvent under reduced pressure, the residue was purified by silica gel chromatography with dichloromethane/methanol (20 : 1, v/v) as eluents, affording probe 1 as a white solid (42 mg, yield 67%). ^1^H- and ^13^C-NMR spectra are shown in Fig. S1 and S2,† respectively. ^1^H NMR (400 MHz, 298 K, CDCl_3_): *δ* 7.68 (d, 1H, *J* = 9.6 Hz), 7.46 (d, 1H, *J* = 8.0 Hz), 7.18 (m, 2H), 6.38 (d, 1H, *J* = 9.6 Hz), 2.08 (s, 3H), 2.05 (s, 3H). ^13^C NMR (100 MHz, 298 K, CDCl_3_): *δ* 160.6, 155.0, 153.3, 143.1, 128.9, 118.8, 116.4, 116.0, 110.5, 24.7, 24.0. HR-ESI-MS: calcd for 1 (C_11_H_12_O_3_PS, [M + H]^+^) 255.0245; found, 255.0249 (Fig. S3†).

### General procedure for F^−^ detection

Unless otherwise noted, the fluorescence of probe 1 (10 μM) reacting with F^−^ was measured in a mixture of DMSO and Tris buffer solution (pH 8.0) (7 : 3, v/v) as follows. The stock solution (1 mM) of probe 1 was prepared by dissolving 1 in DMSO. In a test tube, 3.5 mL of DMSO, 0.5 mL of Tris buffer solution and 50 μL of the stock solution of probe 1 were mixed, followed by adding F^−^ solution. The final volume was adjusted to 5 mL with Tris buffer solution. After incubation at room temperature (25 °C) for 30 min, a 3 mL portion of the reaction solution was transferred to a 1 cm quartz cell to monitor fluorescence with *λ*_ex/em_ = 385/455 nm. Meanwhile, a solution containing no F^−^ (control) was made and recorded under the same conditions for comparison.

### Procedure for determining F^−^ in real samples

To assess the potential use of probe 1 in determining F^−^ in real samples, two water samples were collected from tap water and Yingze Lake (Taiyuan), respectively. Stock solutions of sodium fluoride (1 mM) were prepared in tap water and Yingze Lake water which were pretreated by filtering with 0.22 μm cellulose acetate membranes. The following determination was performed as the aforementioned procedure.

Besides, several commercially available toothpastes were selected to evaluate the practical application of probe 1. Firstly, 0.2 g of toothpaste was accurately weighed and dissolved in 30.0 mL water. Then, the mixture was heated to 70 °C and stirred for 3 h. After cooling down to room temperature, the mixture was centrifuged at 13 000 rpm for 10 min. The supernate was transferred to a 100 mL volumetric flask and the residue was washed three times with water. Finally, the stock solution of toothpaste was prepared by adding water to the scale.

## Results and discussion

### Spectroscopic response of probe 1 to F^−^

**Fig. 1:**
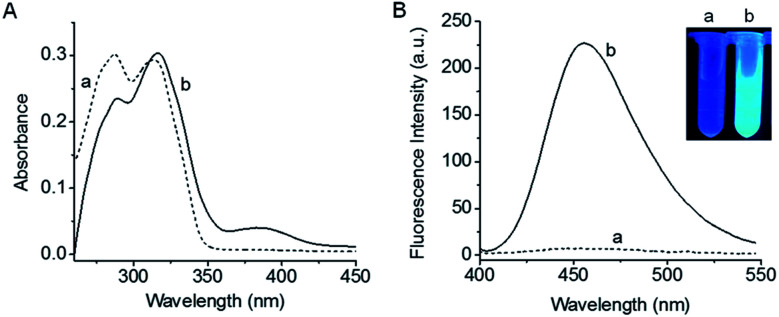
(A) Absorption and (B) fluorescence emission spectra of probe 1 (10 μM) before (a) and after (b) reaction with F^−^ (200 μM) for 30 min in a mixture of DMSO and Tris buffer solution (pH 8.0). *λ*_ex_ = 385 nm.

Absorption and fluorescence spectra of 1 before and after reaction with F^−^ are shown in Fig. 1. As is seen, probe 1 has two absorption peaks at 285 nm and 315 nm. Besides, 1 itself exhibits almost no fluorescence emission due to the blocking of hydroxyl in coumarin,^35–37^ which is rather favourable for the sensitive detection. Upon treatment with F^−^, the maximum absorption peaks of the reaction system are red-shifted and a new absorption weak appears at 385 nm. Meanwhile, a large fluorescence enhancement is produced at 455 nm, accompanied by a distinct fluorescence color change (see the inset of Fig. 1B). Moreover, both the absorption and fluorescence spectra of the reaction system are similar to those of fluorophore coumarin (Fig. S4†), indicating that the reaction of 1 with F^−^ leads to the release of coumarin. The HR-ESI-MS analysis (Fig. S5†) further proves the generation of coumarin (*m*/*z* calcd for [M − 1]^−^ 161.0244, found 161.0243).

The fluorescence kinetics curves of 1 reacting with different concentrations of F^−^ are shown in Fig. S6.† It can be seen that higher concentrations of F^−^ lead to faster reaction velocity and larger fluorescence enhancement, while the fluorescence intensity of probe 1 hardly changes during the same period of time in the absence of F^−^, implying the good stability of 1. Besides, intense fluorescence enhancement can be obtained at the reaction time of 30 min, thus, 30 min was chosen as the response time.^38,39^ Then the effect of pH on the reaction system was studied. As shown in Fig. S7†, the fluorescence signal can be effectively turned on in the pH range of 7.0–9.5, and the maximum fluorescence enhancement appears at pH 8.0. These observations suggest that probe 1 is suitable for biological applications.

**Fig. 2:**
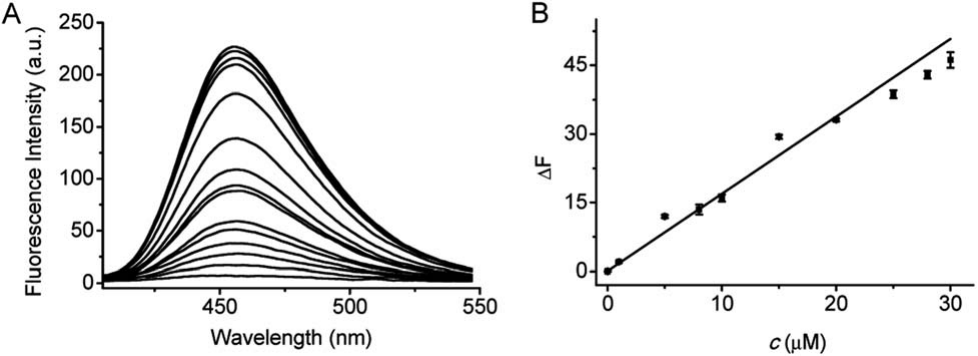
(A) Fluorescence responses of probe 1 (10 μM) to F^−^ at varied concentrations (0, 5, 10, 20, 30, 40, 50, 60, 80, 100, 120, 140, 160, 180, and 200 μM). (B) Linear fitting curve of Δ*F* toward the concentration (*C*) of F^−^ from 0 to 30 μM. *λ*_ex/em_ = 385/455 nm.

Under the above optimized conditions, the fluorescence response of probe 1 to F^−^ at varied concentrations (0–200 μM) is depicted in Fig. 2. The fluorescence intensity is increased by increasing the F^−^ concentration, and a good linearity can be obtained in the concentration range of 0–30 μM with the equation Δ*F* = 1.69 × *c* (μM) + 0.02 (*R* = 0.993), where Δ*F* is the fluorescence difference in the presence and absence of F^−^. The detection limit (3*S*/*m*, in which *S* is the standard deviation of blank measurements, *n* = 11, and *m* is the slope of the linear equation) was determined to be 0.29 μM F^−^. According to the WHO standards, the maximum contaminant level for F^−^ in drinking water is 1.5 mg L^−1^ (about 80 μM).^20^ Therefore, probe 1 can assay F^−^ in drinking water. Notably, prompted by the high sensitivity of probe 1, we have tried to utilize 1 to make fluorescence test strips for F^−^. The test strips are prepared by immersing filter papers (4 × 1 cm^2^) in a mixture of DMSO and Tris buffer solution containing 1 mM of probe 1 and then drying in air. After immersing in the F^−^ aqueous solution for several seconds and drying in air, the fluorescence test strips displays an enhancement in fluorescence signal with increasing the F^−^ concentration. As shown in Fig. S8,† the test strips shows distinct fluorescence when the concentration of F^−^ is as low as 15 μM. Although the sensitivity of the test strip is lower than that of the solution made by probe 1, it is fully competent in the qualitative analysis of F^−^.

**Fig. 3:**
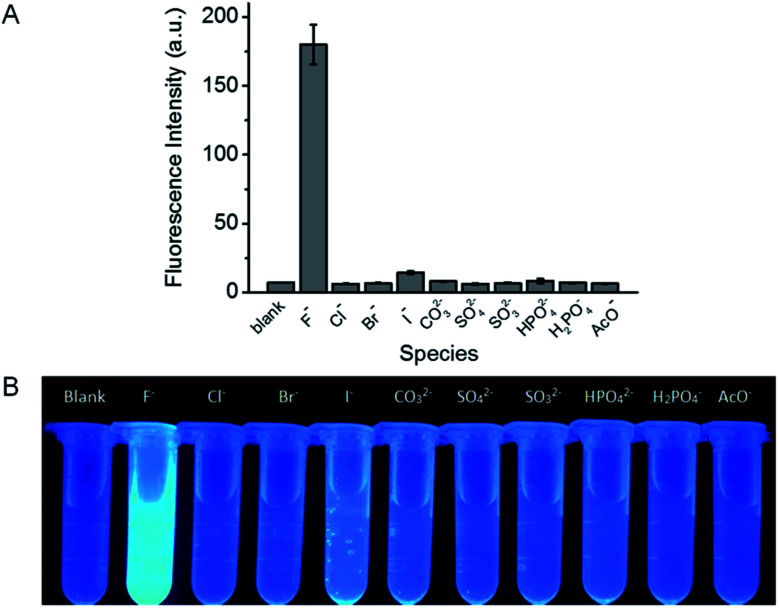
(A) Fluorescence response of probe 1 (10 μM) to various anions (200 μM) in a mixture of DMSO and Tris buffer solution (pH 8.0). The results are the mean ± standard deviation (SD) of three separate measurements. *λ*_ex/em_ = 385/455 nm. (B) Fluorescence images of probe 1 (10 μM) under UV irradiation (365 nm) in the presence of various anions (200 μM).

The specificity of probe 1 towards F^−^ over other common anions, including Cl^−^, Br^−^, I^−^, CO_3_^2−^, SO_4_^2−^, SO_3_^2−^, HPO_4_^2−^, H_2_PO_4_^−^, and AcO^−^ was investigated. At the concentration of 200 μM, only F^−^ induces intense fluorescence enhancement while other species tested scarcely influence the fluorescence of probe 1 (Fig. 3A), indicating that probe 1 demonstrates excellent selectivity for F^−^. Furthermore, the corresponding fluorescence images are clearly showed in Fig. 3B, it can be seen that only F^−^ causes a distinct fluorescence color change and the other anions do not significantly disturb the fluorescence of the reaction solution. Besides, the selectivity of the test strips was also evaluated. As depicted in Fig. S9,† the test strip immersing in F^−^ solution shows strong fluorescence, while the test strips immersing in solutions containing other anions scarcely fluoresce. This observation indicates the high selectivity of the test strips towards F^−^.

### Application of probe 1 to determining F^−^ in real samples

The high sensitivity and selectivity of probe 1 are anticipated to be useful for determining F^−^ in real samples. As mentioned above, fluoride contamination in groundwater happens around the world. Hence, water was selected to evaluate the potential application of probe 1. The real-water samples were collected from tap water and Yingze Lake, respectively and filtered with 0.22 μm cellulose acetate membranes before use. As shown in Fig. S10† and Table 1, F^−^ in both of these two blank samples are not detectable, which may be attributed to the very low concentration of F^−^ (<0.29 μM, the detection limit of probe 1). The recommended limit for class V surface water in China (GB3838-2002) is 1.5 mg L^−1^ (80 μM).^23^ Thus both of the two water samples have not been fluoride contaminated. Moreover, different amounts of F^−^ were spiked into these two real-water samples to minic the contaminated water samples, and the F^−^ contents were determined by probe 1. The results show that the concentrations calculated from our method are in good agreement with those added into the samples, and no significant difference is found at the 95% confidence level using a paired *t*-test.^40,41^ This validates the applicability of probe 1 in determining F^−^ in real-water samples. Encouraged by the assay results in water samples, we further analyze F^−^ contents in commercially available toothpaste samples with probe 1. The results (Fig. S10C† and Table 2) show that the F^−^ contents determined by our method accord well with those marked on the labels of toothpaste, and no significant difference is found at the 95% confidence level.

**Table 1:** Determination of F^−^ concentration (μM) in real-water samples^a^

Sample	No.	F^−^ added (μM)	F^−^ determined (μM)	Recovery (%)
Tap water	1	0	ND^b^	—
	2	20	19.11 ± 0.71	95.6 ± 3.5
	3	25	25.34 ± 0.21	101.4 ± 0.8
Yingze Lake	1	0	ND	—
	2	20	21.40 ± 0.65	107.0 ± 3.3
	3	25	24.38 ± 1.07	97.5 ± 4.3

a Mean of three determinations ± standard deviation.

b Not detectable.

**Table 2:** Determination of F^−^ content in toothpaste samples^a^

Sample	Brand	F^−^ content (marked)	F^−^ content (detected)
1	Jiajieshi	0.11%	0.12% ± 0.01%
2	Heiren	0.10%	0.09% ± 0.02%
3	Fangxianzi	0	ND^b^

a Mean of three determinations ± standard deviation.

b Not detectable.

## Conclusions

In summary, we have developed a highly sensitive and selective fluorescent probe for F^−^ by incorporating the dimethylphosphinothionyl group as a recognition moiety into the fluorophore of coumarin. The probe exhibits an intense fluorescence off–on response to F^−^*via* the fluoride-triggered cleavage of the dimethylphosphinothionyl group, followed by the release of fluorophore. Compared with the existing fluorescent probes for F^−^ detection, probe 1 displays higher sensitivity even in aqueous environment (Table S1†). The probe has been applied to determine F^−^ in real-water samples and commercially available toothpaste samples, and the results are satisfying. The simplicity and excellent performance of probe 1 make it useful in detecting F^−^ in more real-samples.

## Conflicts of interest

There are no conflicts to declare.

## Supplementary Material

RA-009-C9RA06342K-s001
