# Supplementary material for: A novel fluorescent off–on probe for the sensitive and selective detection of fluoride ions
Source: RSC Adv. 2019 Oct 10;9(55):32308–12. doi: 10.1039/c9ra06342k (PMC9073186; doi:10.1039/c9ra06342k)
Supplement: RA-009-C9RA06342K-s001 [file RA-009-C9RA06342K-s001.pdf]

Electronic Supplementary Material (ESI) for RSC Advances.
This journal is © The Royal Society of Chemistry 2019

Electronic Supplementary Information for

A novel fluorescence off-on probe for the sensitive and selective detection of fluoride ions

Lihong Li,*[ab] Min Zhang,[a] Kaijing Chang,[b] Yu Kang,[a] Guodong Ren,[a] Xiaoyu Hou,[a] Wen Liu,[b] Haojiang Wang,[b] Bin Wang[b] and Haipeng Diao*[ab]

[a]*Department of Biochemistry and Molecular Biology, Shanxi Medical University, Taiyuan 030001, PR China*

[b]*School of Basic Medical Sciences, Shanxi Medical University, Taiyuan 030001, PR China*

E-mail: lilh@sxmu.edu.cn, diaohp@sxmu.edu.cn

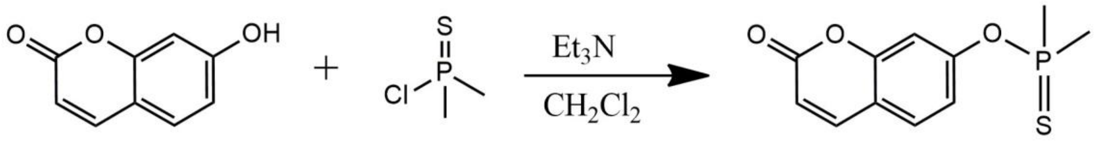

1

Scheme S1. Synthetic route of probe **1**.

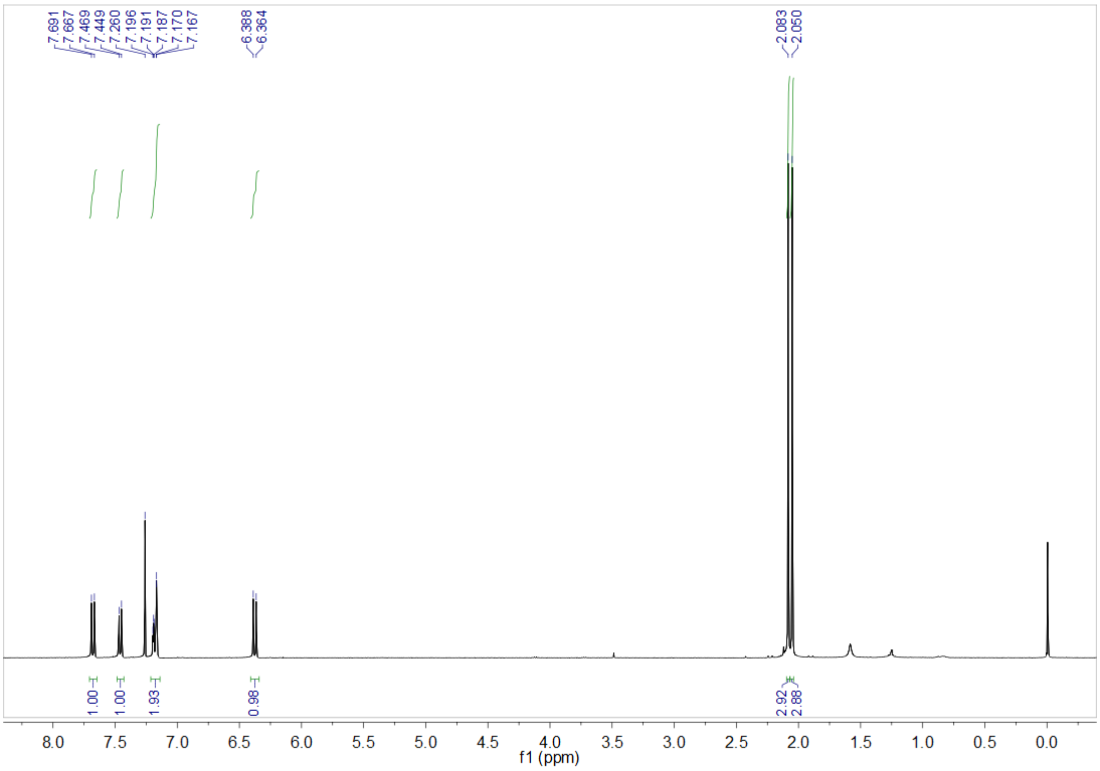

Fig. S1. ^{1}H NMR spectrum of probe **1** (400 MHz, 298 K, $CDCl_3$).

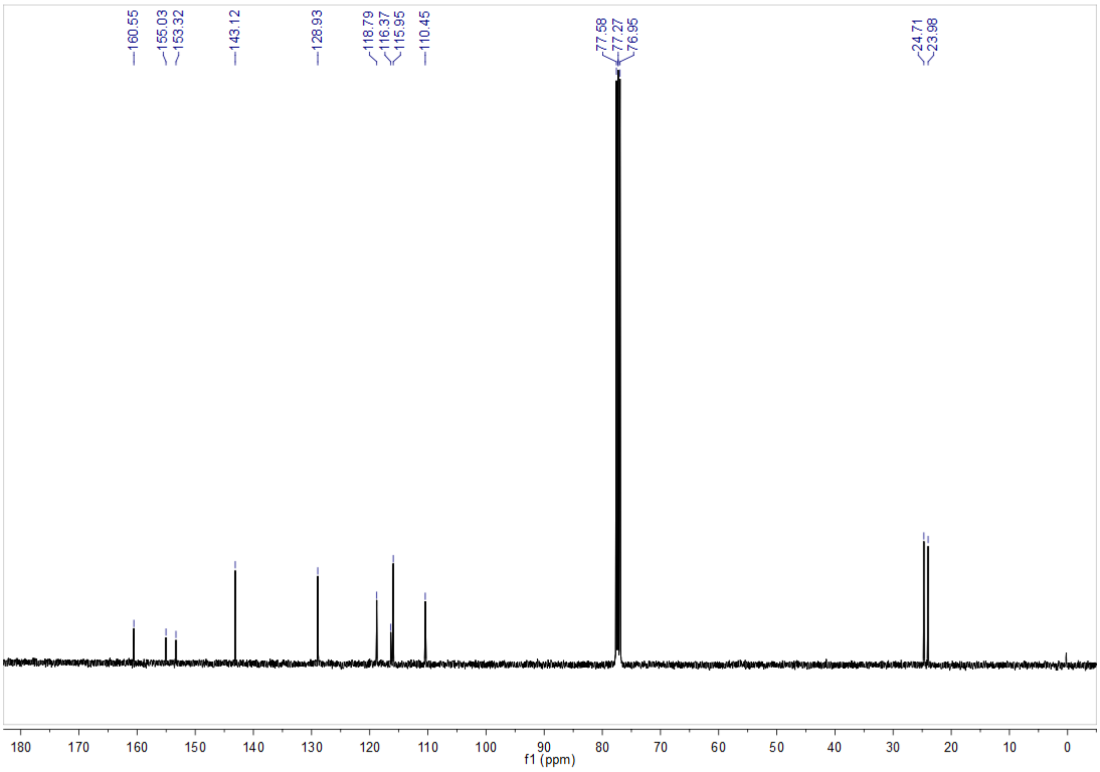

Fig. S2. ^{13}C NMR spectrum of probe **1** (100 MHz, 298 K, $CDCl_3$).

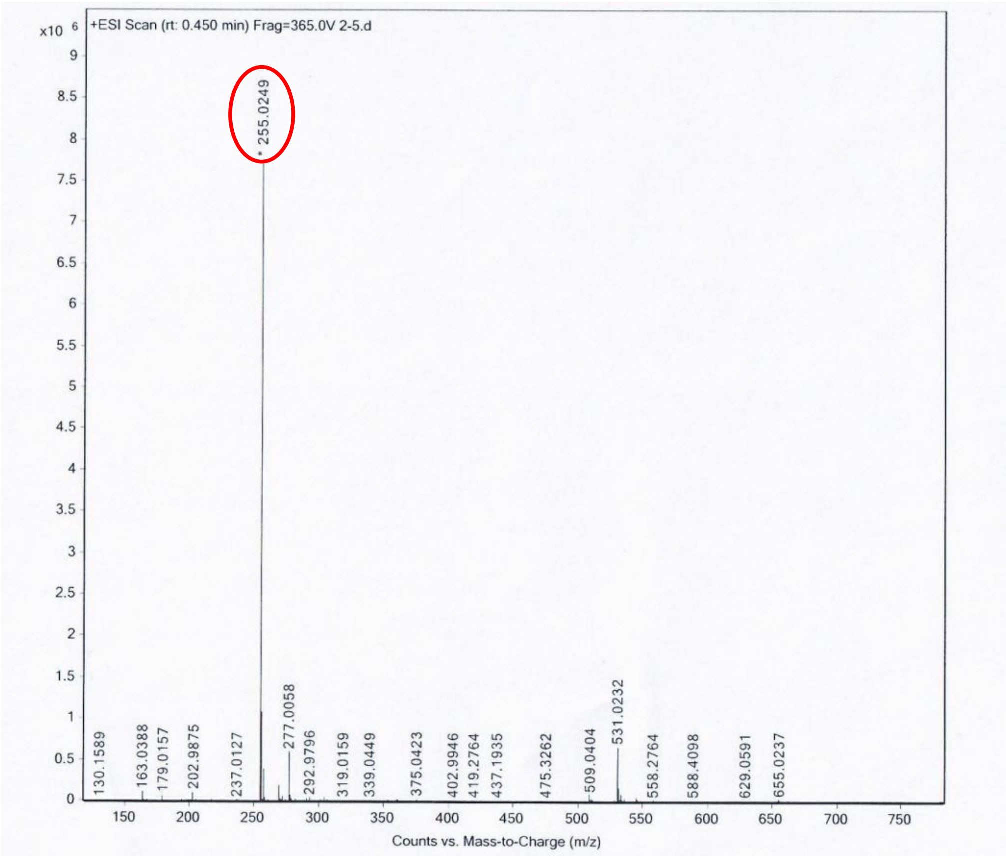

Fig. S3. High resolution ESI-MS of probe **1**.

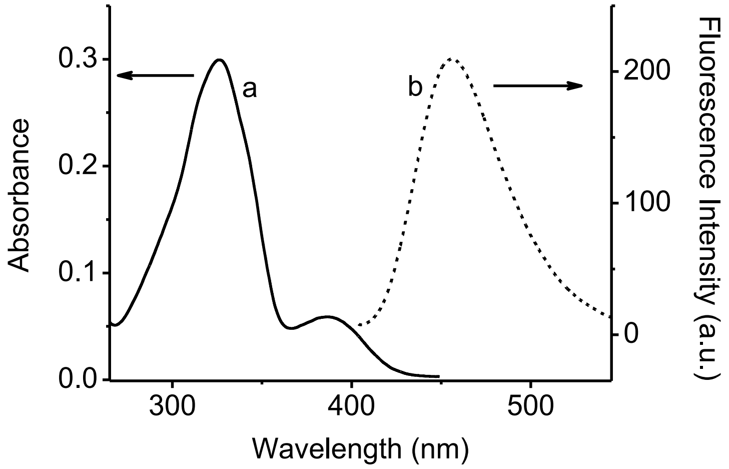

Fig. S4. Absorption and fluorescence emission spectra of coumarin (10 μM) in a mixture of DMSO and Tris buffer solution (pH 8.0).

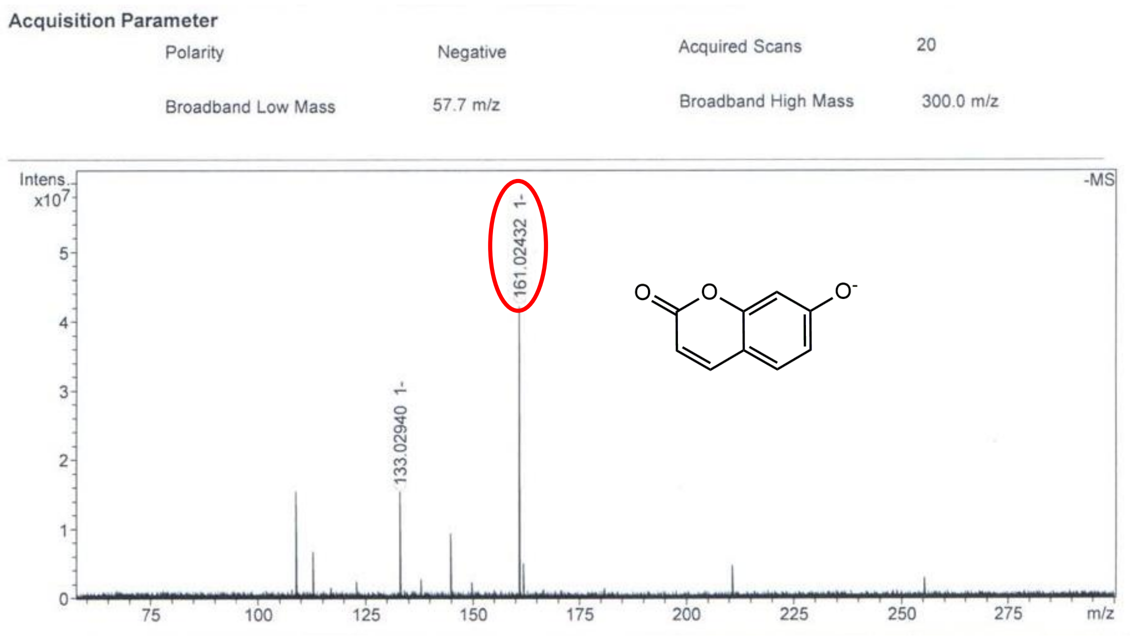

Fig. S5. High resolution ESI-MS of the reaction solution of probe **1** (50 μM) with F^- (500 μM). The peak at *m/z* = 161.0243 reflects the formation of the fluorophore coumarin.

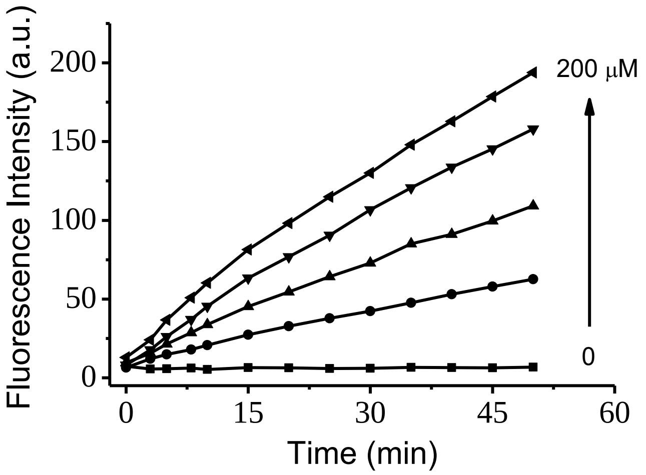

Fig. S6. Plots of fluorescence intensity *vs*. the reaction time of probe **1** (10 μM) with varied concentrations of F^- (0-200 μM). The measurements were performed in a mixture of DMSO and Tris buffer solution (pH 8.0) at room temperature. $\lambda_{ex/em}$ = 385/455 nm.

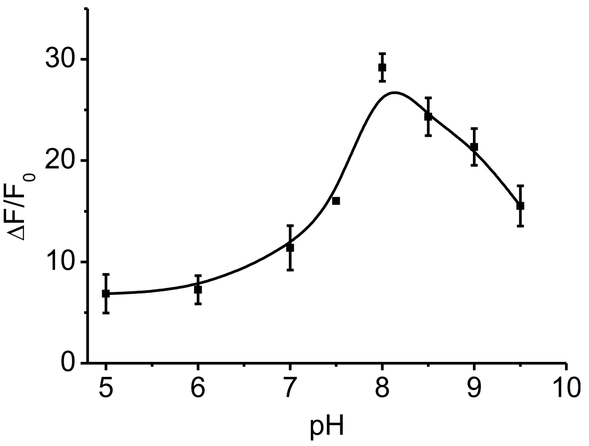

Fig. S7. Effect of pH on the fluorescence ($\lambda_{ex/em}$ = 385/455 nm) of probe **1** (10 μM) reacting with F^- (200 μM). The results are the mean ± standard deviation (SD) of three separate measurements.

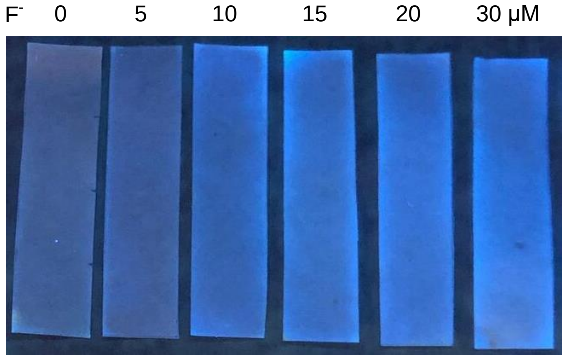

Fig. S8. Fluorescence images of test strips for detecting F^- in aqueous solution with varied F^- concentrations (0, 5, 10, 15, 20, and 30 μM). The test strips were excited at 365 nm by a hand-held UV lamp.

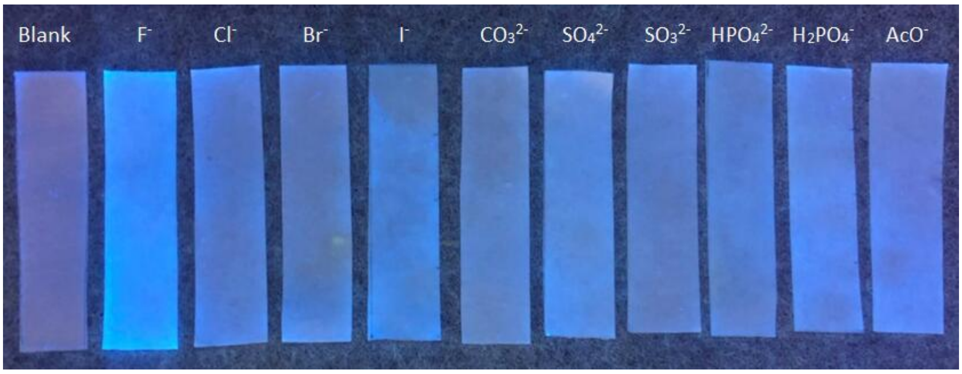

Fig. S9. Fluorescence images of test strips in the presence of various anions (200 μM). The test papers were excited at 365 nm by a hand-held UV lamp.

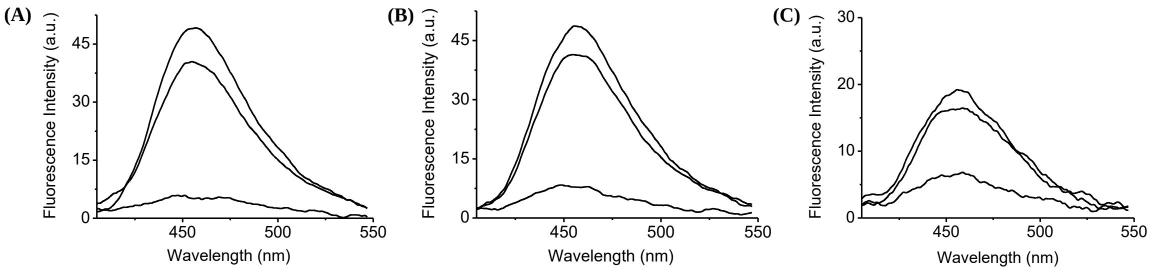

Fig. S10. Fluorescence response of probe **1** (10 μM) to F^- in real samples. (A) Tap water: samples No. 1-3 from bottom to up; (B) Yingze Lake water: samples No. 1-3 from bottom to up; (C) Toothpaste: samples No. 1-3 from up to bottom. $\lambda_{ex/em}$ = 385/455 nm.

Table S1. Comparison of probe **1** with other recently reported fluorescent probes for F^-

Mechanism	Detection limit	Wavelength	Detection media	Literature
P-O cleavage	0.29 μM	λ_{em} = 455 nm	DMSO/Tris buffer (7:3, v/v)	This work
H-bond interaction	Not mentioned	λ_{em} = 495 nm	CH_3CN	Zhou *et al.*, *Spectrochim. Acta A*, 2018, **204**, 777
H-bond interaction	3.2 μM	λ_{em} = 510 nm	toluene	Lu *et al.*, *Sens. Actuators B*, 2018, **270**, 291
Deprotonation of N-H proton	0.28 μM	λ_{em} = 598 nm	DMSO	Yang *et al.*, *Sens. Actuators B*, 2015, **210**, 784
Si-O cleavage	0.59 μM	λ_{em} = 564 nm	CH_3CN/HEPES buffer (8:2, v/v)	Yoo *et al.*, *RSC Adv.*, 2016, **6**, 19910
Si-O cleavage	3.5 μM	λ_{em} = 480 nm	ethanol	Zhang *et al.*, *Anal. Bioanal. Chem.*, 2017, **409**, 2075
Si-O cleavage	1.03 μM	λ_{em} = 523 nm	DMSO	Roy *et al.*, *Chem. Coummun.*, 2014, **50**, 5510
Si-O cleavage	0.17 μM	λ_{em} = 485 nm	CH_3CN	Zhang *et al.*, *Chem. Commun.*, 2014, **50**, 14021

Si-O cleavage	Not mentioned	λ_{em} = 589 nm	CH_3CN/H_2O (1:1, v/v) or CH_3CN	Kim *et al.*, *Org. Lett.*, 2007, **9**, 3109
Si-O cleavage	85 nM	λ_{em} = 718 nm	DMSO/H_2O (95:5, v/v)	Cao *et al.*, *Tetrahedron Lett.*, 2012, **53**, 2107
Si-O cleavage	0.1 μM	λ_{em} = 682 nm	CH_3CN	Xie *et al.*, *J. Fluoresc.*, 2016, **26**, 1737
Si-O cleavage	0.12 μM	λ_{em} = 676 nm	CH_2Cl_2	Cao *et al.*, *RSC Adv.*, 2012, **2**, 418
Si-O cleavage	17.2 μM	λ_{em} = 493 nm	DMF/HEPES buffer (7:3, v/v)	Zhou *et al.*, *Dyes Pigments*, 2018, **158**, 277
P-O cleavage	9.8 nM	λ_{em} = 536 nm	DMSO/Tris buffer (7:3, v/v)	Kim *et al.*, *Dyes Pigments*, 2015, **112**, 170
P-O cleavage	48 nM	λ_{em} = 669 nm	DMSO/Tris buffer (7:3, v/v)	Du *et al.*, *Anal. Chim. Acta*, 2018, **1030**, 172.
